# Parenting concerns among patients with cancer: a qualitative meta-synthesis

**DOI:** 10.3389/fpsyg.2026.1801763

**Published:** 2026-09-03

**Authors:** Yuchen Jiao, Ping Zhu, Liuliu Zhang, Bing Wu

**Affiliations:** 1 Medical Oncology, Jiangsu Cancer Hospital & The Affiliated Cancer Hospital of Nanjing Medical University, Nanjing, China; 2 Nursing Department, Jiangsu Cancer Hospital & The Affiliated Cancer Hospital of Nanjing Medical University, Nanjing, China

**Keywords:** cancer patients, experience, meta-synthesis, parenting concern, qualitative study

## Abstract

**Objectives:**

This qualitative meta-synthesis aimed to interpret how cancer patients experience and manage parenting concerns, and to identify implications for family-centered psychosocial care.

**Design:**

A qualitative meta-synthesis was conducted.

**Methods:**

PubMed, Embase, Web of Science, Cochrane Library, CINAHL, EBSCO, ProQuest, China Science and Technology Journal Database, China National Knowledge Infrastructure, and Wanfang Database were searched from inception to April 2025. Qualitative studies published in Chinese or English were included. The quality of included studies was assessed using the Joanna Briggs Institute (JBI) Critical Appraisal Checklist for Qualitative Research. Data were synthesized using thematic synthesis.

**Results:**

Thirty-seven studies were included. Three analytical themes were generated: disrupted parental identity and layered threat appraisal under cancer-related uncertainty; protective parenting and family adaptation as relational resources; and unmet multidimensional needs for family-centered and developmentally tailored care. Together, these themes showed that parenting concerns among cancer patients involved not only emotional distress and perceived threats to children, but also protective efforts, meaning-making, family adaptation, and unmet needs for practical, communicative, informational, and psychosocial support.

**Conclusion:**

Parenting concerns among cancer patients represent a dynamic, relational process in which cancer-related uncertainty disrupts parental identity, activates layered threat appraisals about children and family continuity, and may also mobilize protective parenting and family adaptation. These findings suggest that oncology care should move beyond individual distress management and incorporate routine assessment of parenting concerns, developmentally appropriate parent–child communication support, practical caregiving assistance, family-level psychosocial referral, and genetic counseling when hereditary risk is relevant.

**Systematic review registration:**

https://www.crd.york.ac.uk/PROSPERO/view/CRD42023424201, identifier CRD42023424201.

## 1 Introduction

Cancer remains a major global public health challenge. According to the GLOBOCAN 2022 estimates, nearly 20 million new cancer cases occurred worldwide in 2022, with China representing one of the countries with a high cancer burden (Bray et al., 2024). At the same time, the incidence of early-onset cancer has increased markedly over recent decades, meaning that more patients are diagnosed during life stages characterized by active work, partnership, and parenting responsibilities (Zhao et al., 2023). For patients raising dependent children, cancer is therefore not only an individual health crisis but also a family event. Population-based evidence suggests that a considerable number of patients with cancer have minor or dependent children, and earlier estimates from the United States indicated that approximately 1.5 million cancer survivors were raising minor children, with 2.85 million children living with a parent affected by cancer (Weaver et al., 2010).

Parenting concerns refer to the worries and distress experienced by patients with cancer regarding the impact of their illness on their children and their ability to fulfill parenting responsibilities (Hymovich, 1993; Muriel et al., 2012). These concerns may involve practical caregiving, emotional availability, communication about diagnosis and prognosis, children’s psychological adjustment, future care arrangements, and the parenting capacity of a spouse or co-parent (Inhestern et al., 2016a; Muriel et al., 2012). Evidence indicates that parenting concerns are associated with psychological distress and may influence patients’ quality of life, family functioning, and even treatment-related decision-making (Check et al., 2017; Inhestern et al., 2016b; Jewett et al., 2020). From a family-centered care perspective, these concerns should not be regarded as private family issues alone, but as clinically relevant supportive care needs that require recognition within oncology care (Inhestern et al., 2021). In Chinese and other East Asian contexts, cultural expectations around family responsibility, child protection, and interdependence may further shape how patients interpret their parental role and communicate illness-related information to children (Mori et al., 2023).

Despite the clinical significance of parenting concerns, the needs of patients with cancer who are parents remain insufficiently addressed in routine oncology practice. Recent qualitative and mixed-methods studies have shown that many parents want guidance on how to talk with their children, maintain family normality, and balance treatment demands with parenting responsibilities. However, healthcare professionals do not always proactively identify or respond to these family-specific concerns (Inhestern et al., 2016a). Interventions focusing on parent-child illness communication and family support are emerging, but the evidence base remains limited, and effective, feasible, and culturally adaptable approaches have not yet been fully established (Zhao et al., 2024).

Existing reviews have contributed important knowledge by summarizing the experiences of parents with cancer, the impact of parental cancer on children, and the broader challenges of parenting during cancer (Matuszczak-Swigon and Bakiera, 2021). However, three gaps remain. First, previous syntheses have mainly described parenting difficulties and support needs, whereas the processes through which cancer-related disruption threatens parental identity, generates layered concerns about children, and activates protective parenting have not been sufficiently theorized. Second, the cultural and family-centered meanings of parenting concerns, particularly evidence from Chinese-language studies and East Asian family contexts, have not been explicitly integrated. Third, the implications of these experiences for tailored assessment, parent-child communication guidance, and family-level psychosocial support remain insufficiently specified. Therefore, this qualitative meta-synthesis aimed to integrate Chinese and English qualitative studies on parenting concerns among patients with cancer and to develop an interpretive understanding of how these concerns are experienced, constructed, and managed within cancer-affected families. The findings may inform the identification of high-need families and the development of family-centered, developmentally appropriate supportive care.

## 2 Methods

### 2.1 Synthesis methodology

Meta-synthesis is one of the most commonly used methods in systematic reviews of qualitative research. It enables the aggregation of qualitative data in a specific field to gain an in-depth understanding and interpretation of the essence of a phenomenon, forming new explanations, accumulating knowledge, and developing new theories (Walsh and Downe, 2005). This study utilized meta-synthesis not to simply summarize the original qualitative studies, but rather as an “interpretive integration” of parenting concerns from the perspective of cancer patients. This method facilitates a more comprehensive and in-depth understanding of cancer patients’ views, experiences, and coping strategies regarding parenting issues. This review employed the integration analysis framework of “Thematic Synthesis” (Thomas and Harden, 2008), which is based on the critical realist philosophy by Thomas and Harden. This framework is effective for addressing questions concerning people’s views and experiences (Harden et al., 2004; Thomas et al., 2004). We conducted iterative meta-data analysis, involving continuous discussion and abstraction, to generate new perspectives and insights regarding the experiences of parenting concerns among cancer patients. This study was conducted in accordance with the reporting guideline “Enhancing Transparency in Reporting the Synthesis of Qualitative Research: ENTREQ” (Tong et al., 2012).

### 2.2 Search methods

The search strategy was developed *a priori* according to the Participants, Phenomenon of Interest, and Context (PICo) framework (Lockwood et al., 2015). Specifically, the participants were cancer patients with children; the phenomenon of interest was the experiences of cancer patients with children simultaneously facing parenting concerns; the context was that cancer and its treatment trigger parenting concerns in patients.

Based on the PICo framework, three groups of search concepts were used: (1) cancer, including terms such as cancer, neoplasm, carcinoma, tumor, malignancy, and malignant; (2) parenting concerns and parenting, including terms such as parenting concern, parenting stress, parenting worries, parental concern, parenting, raising, rearing, nurturing, and child; and (3) qualitative research, including terms such as qualitative research, interview, focus group, grounded theory, phenomenology, ethnography, narrative analysis, field study, and participant observation. Both controlled vocabulary terms and free-text terms were used where applicable, and Boolean operators were used to combine terms within and across the three concept groups.

The following English and Chinese databases were searched from inception to April 2025: PubMed, Embase, Web of Science, Cochrane Library, CINAHL, EBSCO, ProQuest, China Science and Technology Journal Database, China National Knowledge Infrastructure, and Wanfang Database. The search strategy was reviewed by a researcher with experience in evidence-based practice before the formal search was conducted. An example PubMed search strategy is provided in the main text to enhance reproducibility, and the complete search strategies for all databases are provided in Supplementary Material 1.

Example PubMed search strategy

(cancer OR neoplasm* OR carcinoma* OR tumor* OR tumour* OR malignan*) AND (“parenting concern*” OR “parenting stress” OR “parenting worries” OR “parental concern*” OR parenting OR parent* OR raising OR rearing OR nurturing OR child*) AND (“qualitative research” OR interview* OR “focus group*” OR phenomenolog* OR ethnograph* OR “grounded theory” OR “narrative analysis” OR “field stud*” OR “participant observation”).

Only peer-reviewed articles published in Chinese or English were included. This language restriction was applied because the review team had bilingual expertise in Chinese and English and aimed to integrate evidence from both international and Chinese research contexts.

### 2.3 Study selection

All retrieved records were imported into EndNote X9 software (Thomson Reuters, Philadelphia, PA, USA) for management and duplicate removal. Then, two researchers with evidence-based experience (Yuchen Jiao and Bing Wu) independently screened titles and abstracts according to the inclusion and exclusion criteria (Table 1). Following this, two researchers further reviewed the full texts to determine the final included studies. During the study selection process, all discrepancies were resolved through weekly research team meetings involving all authors and frequent communication, ultimately reaching consensus.

**TABLE 1:** Inclusion and exclusion criteria of literature.

Eligibility component	Inclusion criteria	Exclusion criteria
Participants	Patients diagnosed with cancer including any stage of cancer; Cancer patients have at least one child with no age restriction	No additional exclusion criterion
Phenomena of interest	Experiences of parenting concern in cancer patients: the predicament of a population facing dual role: parent and patient	No additional exclusion criterion
Context	After the parents were diagnosed with cancer	No additional exclusion criterion
Design and publication type	All types of qualitative research, and the qualitative parts of mixed research that can extract data	Non-peer-reviewed publication (e.g., editorial, abstracts, commentary, and thesis); Articles in non-Chinese or non-English

### 2.4 Quality appraisal

The quality of the included studies was assessed using the JBI Critical Appraisal Checklist for Qualitative Research (The Joanna Briggs Institute, 2017). This checklist comprises 10 items assessing methodological congruity, data collection, data analysis, interpretation of findings, representation of participants and their voices, researcher positioning and influence, ethical considerations, and the link between data analysis and conclusions. Two researchers independently appraised all included studies. Disagreements were discussed and resolved through consensus; when necessary, the full research team was consulted. To enhance transparency, item-level appraisal results were reported to show the methodological strengths and limitations of the included studies. No study was excluded solely on the basis of the quality appraisal results, because all included studies met the eligibility criteria, addressed the review question, and contributed relevant qualitative data to the synthesis. Methodological limitations identified during appraisal, particularly incomplete reporting of researcher reflexivity or researcher influence, were considered when interpreting the findings and assessing the credibility of the synthesis.

### 2.5 Data extraction and analysis

To facilitate qualitative synthesis, one researcher extracted data from the included studies using a standardized extraction form, and another researcher checked the extracted information for accuracy and completeness. Extracted information included first author, publication year, country or region, participant characteristics, cancer type and disease context, study aim, qualitative design, data collection method, data analysis method, phenomenon of interest, and main findings or themes.

Thematic synthesis was conducted following the approach proposed by Thomas and Harden (Thomas and Harden, 2008). First, findings and relevant participant quotations from the primary studies were coded line by line. Second, similar codes were compared, grouped, and organized into descriptive themes. Third, through repeated comparison, discussion, and abstraction, analytical themes were developed to move beyond a descriptive summary of primary studies. NVivo software was used to assist the coding and organization of qualitative data.

To enhance interpretive rigor, two researchers independently coded the data and then compared coding results. Differences were discussed in regular team meetings until consensus was reached. During synthesis, we repeatedly returned to the original studies to ensure that the analytical themes were grounded in the primary data while also providing a higher-level interpretation of parenting concerns among cancer patients. The quality appraisal results were considered during interpretation, especially when findings were primarily supported by studies with incomplete methodological reporting.

### 2.6 Researcher reflexivity

Researcher reflexivity was considered throughout the review process. The review team included researchers with backgrounds in oncology nursing, palliative care, psychosocial care, evidence-based practice, and qualitative research. These professional backgrounds facilitated sensitivity to the clinical and family-centered meanings of parenting concerns, but they may also have shaped our attention to supportive care needs and family adaptation.

To reduce the influence of pre-existing assumptions, several strategies were used. First, screening, quality appraisal, coding, and theme development were conducted by at least two researchers independently. Second, researchers kept analytic notes during coding and synthesis to record emerging interpretations, uncertainties, and potential assumptions. Third, disagreements were discussed in regular team meetings, and alternative interpretations were considered before final themes were agreed upon. Fourth, the team repeatedly checked the analytical themes against the original studies to ensure that the synthesis remained grounded in the primary qualitative evidence. These procedures were used to strengthen reflexivity, transparency, and credibility in the synthesis process.

## 3 Results

### 3.1 Record retrieval results

**FIGURE 1:**
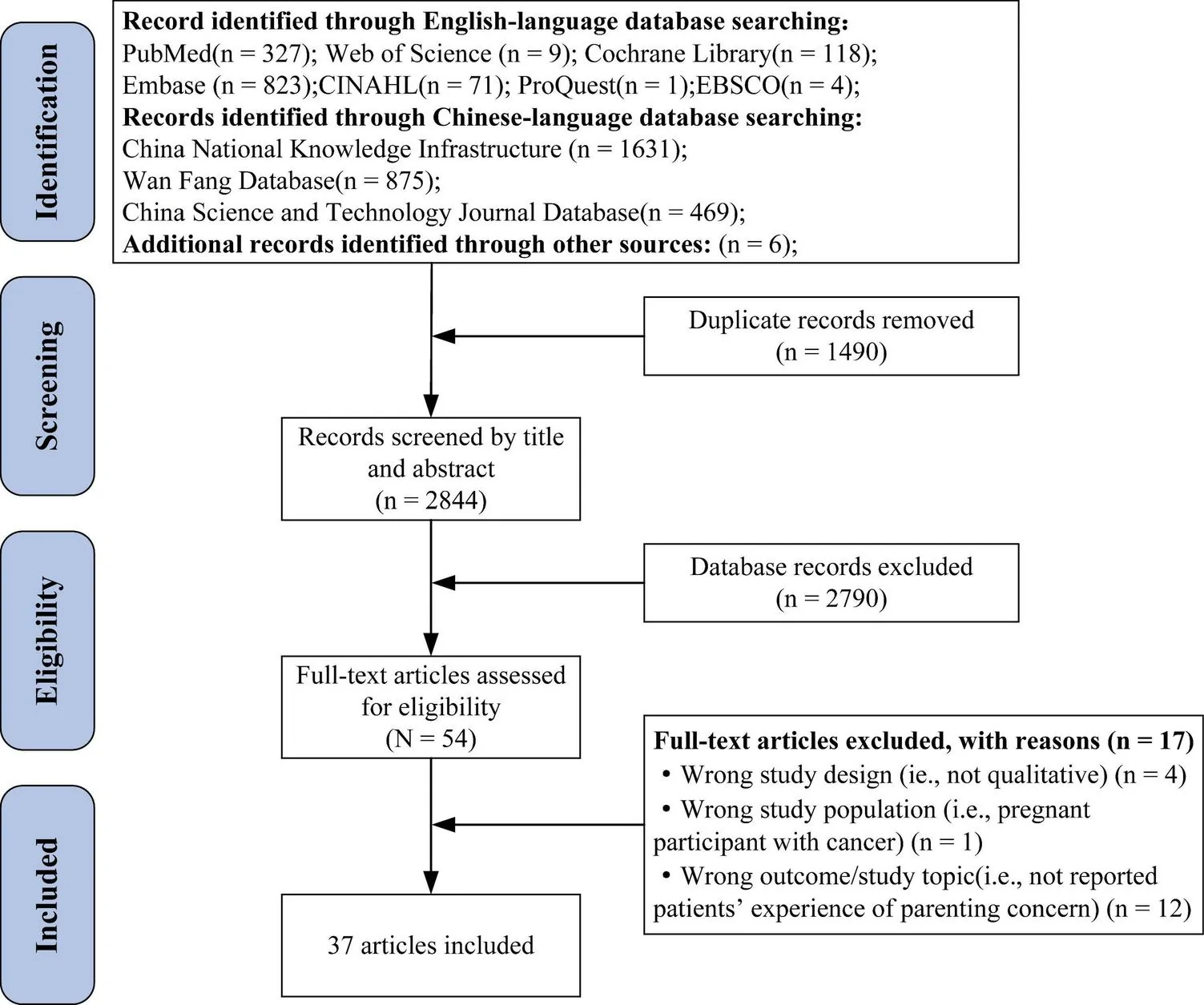
Flowchart of search results and study selection.

A total of 4,328 articles were identified through database searches, and 6 articles were identified through other sources. After removing 1,490 duplicate documents, 2,844 articles remained. After reading the titles and abstracts of the remaining 2,844 articles, 2,790 articles were excluded. Then, we read the full texts of 54 articles, 17 were excluded for the following reasons: research designs did not meet the criteria (such as non-qualitative studies, secondary literature reviews, etc.); study populations did not meet the criteria (such as including pregnant women); and research topics or final findings did not meet the criteria (i.e., articles not focusing on the parenting concerns experienced by cancer parents with minor children, but rather other thematic studies). In the final review, 37 studies (3 Chinese literature and 34 English literature) met the inclusion criteria and were included in the quality assessment (Figure 1).

### 3.2 Study characteristics and quality

Table 2 shows an overview of the characteristics of included studies. All included studies were published between 2000 and 2025, with 43.2% published in the last 5 years (*N* = 16). Studies were mainly conducted in the United States (*N* = 12) and China (*N* = 7). The included studies explored the experiences of cancer patients raising children from various aspects, such as disclosing cancer diagnosis to children, navigating parent-caregiver role conflicts and ethical issues, challenges in patient-child interactions, and the support needs of patients in raising their children. The sample sizes of the 37 included studies ranged from 6 to 224 cancer patients, with different studies having varying criteria on the age of children and the time of diagnosis. Various qualitative designs were adopted in the included studies. The most commonly used qualitative method was the phenomenological approach (*N* = 9), followed by the focus group method (*N* = 6), grounded theory research (*N* = 5), descriptive qualitative research (*N* = 3). One study termed its research report an “interpretive qualitative study”, and 13 studies did not specify their research design. Semi-structured interviews were the most common method for data collection. In terms of data analysis, content analysis (*N* = 11), topic analysis (*N* = 5), Colaizzi analysis (*N* = 4), and grounded theory analysis (*N* = 3) were used. 10 studies only described data analysis or coding steps. There were four studies that conducted data analysis or coding under the guidance of a theoretical framework. Regarding methodological quality, all 37 studies were appraised using the JBI Critical Appraisal Checklist for Qualitative Research, and the item-level appraisal results are presented in Table 3. Overall, most studies demonstrated methodological congruity among research objectives, qualitative methods, data collection, data analysis, and interpretation of findings, and participants’ voices were generally adequately represented. The most common methodological limitation was incomplete reporting of researcher positioning and the influence of the researcher on the research process. These limitations were considered when interpreting the synthesis findings.

**TABLE 2:** Summary of the included studies.

No.	Author, year	Country	Aim	Participants	Methods (Research design, data collection, data analysis)	Findings/themes
1	Romare Strandh et al., 2025	Sweden	To explore parenting concerns and challenges among parents with cancer who were caring for dependent children younger than 18 years	*N* = 22, adults given a diagnosis of any type of cancer who were also a caregiver to at least 1 child younger than 18 years	● A qualitative design ● Semistructured interviews ● Thematic analysis	● Navigating dual roles as a parent with cancer ● Impact of Cancer on Parenting ● Impact on Everyday Family Life
2	Pholsena et al., 2024	USA	To describe patient-reported concerns about their adolescent and how they responded to their adolescent’s behavior	*N* = 6, adolescent-rearing parents with Stage IV cancer	● Not reported ● Single occasion interviews ● Inductive content analysis	Being There without Taking Over ● Struggling to Read My Child ● Attempting to Talk with My Child about My Cancer ● Trying to Maintain Optimism and Understanding My Child
3	Johannsen et al., 2023	Germany	To explore patients’ perspectives on their experiences regarding family-centeredness in cancer care	*N* = 18, cancer patients parenting at least one minor child (<18)	● Qualitative study ● Semi-structured interviews by telephone ● Qualitative content analysis	● Characteristics of (in)adequate family-oriented cancer care and unmet supportive care needs ● Profession-related differences ● Barriers and facilitators for the integration of parenting issues ● Wishes regarding future care
4	Román-Oyola et al., 2022	Puerto Rico (USA)	To explored play experiences between mothers who had completed, or were receiving treatment for breast cancer and their young children and disease-related factors influencing those experiences	*N* = 6; mother were 21 years or older; receiving adjuvant treatment at the time of recruitment or had completed their cancer treatment no more than 5 years before recruitment; had at least one child between 2 years and a half and 6 years, 11 months while receiving treatment	● An interpretative phenomenological study ● Semi-structured interviews ● Guided by constant comparative method including two cycles of coding as described by Saldaña (2013)	● “Changes and challenges,” which contextualized disease-related factors affecting the maternal role ● “Play and relationships with children” described play moments and how treatment affected these
5	Zeng et al., 2023	China	To explore the psychological experiences of cancer patients in informing their minor children of the disease diagnosis	*N* = 14, Cancer patients; The patient has minor children (6–17 years old); Exclude patients with advanced cancer	● Phenomenological research ● Semi-structured interviews ● Colaizzi analysis method	● Reasons for choosing not to disclose ● Many difficulties faced in concealing the illness ● Deliberation before disclosure ● Perceived benefits after disclosure ● Difficulties encountered after disclosure
6	Tavares et al., 2022	Portugal	To identify parenting challenges in families with maternal cancer and develop a psychological intervention program for mothers with BC accordingly to their needs	*N* = 18, women diagnosed with breast cancer, with at least one dependent child at the diagnosis stage (i.e., aged less than 18 years old). Women diagnosed with cancer for less than 12 months and still undergoing oncological treatment (except hormone therapy) are excluded	● Not reported ● Focus groups ● Content analysis method	● Contents of the intervention ● Potential benefits of group intervention ● Structure of the intervention ● Other types of support
7	Chin and Chen, 2023	China	To investigate the support needs identified by Taiwanese breast cancer diagnosed mothers for themselves and their 6–12-year-old children	*N* = 20; mothers who had been diagnosed with breast cancer in the last 2 years; having at least one child between 6 and 12 years old at the time of diagnosis	● A qualitative descriptive study ● Semi-structured individual interviews ● Qualitative content analysis	● Learning to reach out to children ● Dealing with negative emotions ● How to say goodbye to children ● Resetting for the future ● Emotional and health education ● Getting along with a sick mother ● Preparing for uncertainty
8	Zhu et al., 2022	China	To uncover the nuances of the interactive challenges with adolescent daughters from breast cancer-afflicted mothers’ perspective	*N* = 21; mothers with breast cancer female patients; currently raising daughters between ages 13 and 18	● A phenomenological research design ● Semistructured interviews ● Thematic analysis	● Mothers are lost in chaos (inability to handle the shock of cancer, feelings of powerlessness about the uncertainty of their life span, and confusion about how to respond to daughter’s curiosity) ● Mothers struggle to maintain balance (torn between protecting daughters and letting them be independent, and making a tough choice between being a mother or a patient) ● Mothers are immersed in guilt (increasing daughters’ risk of cancer, influencing daughters’ development, and imposing burdens on daughters)
9	Hauskov Graungaard et al., 2023	Denmark	To explore the difficulties parents face when understanding their children’s reactions to parental cancer and parents’ reactions to their children’s perceived needs	*N* = 18, cancer diagnosed more than 6 months earlier and children aged 0–15 years	● Qualitative research ● Semi-structured interview ● Inductive analysis with systematic text condensation	● Understanding the child’s reactions ● Worry and hope about the future ● Normalcy and distraction as coping strategies ● Support experiences and unmet needs in the family
10	Mazaheri et al., 2021	Iran	To explore the perceived threats to the parenting role and opportunities for growth among Iranian mothers with breast cancer	*N* = 20, mothers with diagnosed with breast cancer who had a child younger than 16 years of age	● A qualitative study ● Semi-structured interviews ● Content analysis method	● Threats to parenting role ● Burden on children’s Life ● Turning threats into opportunity: moving toward reorganization
11	Tamura et al., 2021	Japan	To explore the experiences of Japanese fathers with cancer	*N* = 24; adult men undergoing cancer treatment and raising minor children (aged ≤ 19 years)	● The qualitative design based on grounded theory ● Semi-structured interviews; ● Grounded theory approach	“Transformed identity: cancer made me into a father” ● Being swallowed by a torrent of cancer ● Recognizing they are not the fathers they thought they were ● Realizing that time is limited ● Experiencing cancer’s effect on children ● Noticing fathers and children need each other ● Placing more value on time with children than on work ● Being assailed by emotional turmoil that is difficult to handle alone ● Nurturing children like they would not be able to do in the future
12	Park et al., 2021	USA	To describe the challenges, approaches, and decisions related to discussing prognosis among a sample of mothers with metastatic cancer	*N* = 224, patients were self-identified adults with metastatic cancer (defined as stage IV solid tumors with distant metastases per the American Joint Committee on Cancer’s staging system) who had at least one child ≤18 years old	● A mixed research based on web, including descriptive qualitative research ● Structured interviews ● Qualitative content analysis	● Commitment to honesty and protection ● Child developmental readiness ● Beliefs about the right time ● Approaches to discussing prognosis included total honesty, using the language of chronic illness, gradual disclosure, waiting for questions, and emphasizing hope, love, and reassurance
13	Lundquist et al., 2020	USA	To reveal the participants’ roles as mothers while living with advanced breast cancer	*N* = 12, young adult women living with advanced breast cancer	● Hermeneutic phenomenologic method ● Semistructured interviews ● Line-by-line coding and hermeneutic analysis	I’m still Mom ● Being Mom Is Hard ● Time Is Short ● It’s Not Easy for My Kids
14	Chin et al., 2021	China	To examine Taiwanese mothers’ experiences of and cultural practices embedded in parenting young children while in treatment for breast cancer	*N* = 16, mothers with breast cancer; at least 1 child younger than 12 years	● Not reported ● Semistructured individual interviews ● A qualitative content analysis	● Maternal limited disclosure, complementary with children’s tacit knowledge ● The scar is no longer a scar but a symbol for intimate bonding ● Issues of maternal absence for young children and school-aged children ● The power of ‘We are a family’ ● To live a simple life and to live for one’s self
15	Huang et al., 2020	China	To explore the real parenting experiences of breast cancer patients raising underage children	*N* = 16, female breast cancer patients;Cancer diagnosis within 2 years; raising minor children 7–17 years old	● Phenomenological research ● Semi-structured interviews ● Colaizzi analysis method	● Concerns about children ● Communication difficulties ● Protecting children ● Strength from children
16	Steiner et al., 2019	Australia	To explore hospital-based parenting support delivery from patient and co-parent perspectives in context to their parenting experience and support needs	*N* = 12, adult patients with incurable end-stage cancer (any type) who were accessing noncurative hospital treatment and parenting one or more children aged up to 18 years old	● Descriptive qualitative research ● Semi-structured in-depth interviews ● Thematical analysis	● Parenthood during IESC phase: the lived experience and supportive care needs ● Parenting-focused supportive care utilization
17	Loggers et al., 2019	USA	To investigated Hispanic mothers’ willingness to communicate with dependent children about her actual or hypothetical advanced cancer diagnosis	*N* = 9, two focus groups (*N* = 6 participants) and three, one-on-one interviews (*N* = 3); patients with a cancer diagnosis of any stage within the last 2 years, having a child 5 to 12 years old living in the household	● Not reported ● Focus groups and individual interviews ● Content analysis	Participants reported their perceived concerns, parenting challenges and openness to discussing an incurable cancer diagnosis with a dependent child. Audio files were transcribed into English and qualitatively coded using content analysis ● Prior considerations ● Parenting challenges ● Concerns for their children ● Confidence the children will be okay ● Hopes and expectations for their children ● Advanced cancer as an opportunity for positive change ● Communication with their child(ren) about advanced cancer
18	Sinclair et al., 2019	Australia	To explore the communication and resource needs of mothers diagnosed with breast cancer treated with curative intent in communicating with their young children and to identify gaps in the resources and support provided to these women	*N* = 23, 13 mothers who were diagnosed with breast cancer while parenting a young child (age 3–12 years), and 10 health professionals with experience of providing psychosocial support to adults with cancer	● Not reported ● Semi−structured telephone interviews ● The Framework Method for data analysis	● Communication with children about cancer ● Communication resource needs
19	Arida et al., 2019	USA	To describe the experience of mothers with ovarian cancer, elucidating the interaction between their roles as mothers and patients with cancer	*N* = 17, mother (≥18 years old) with a diagnosis of ovarian cancer	● Qualitative research ● Focus groups and individual interviews ● Descriptive coding and framework analysis	● Themes reflected participants’ evolving self-identities from healthy mother to cancer patient to woman mothering with cancer ● Sub-themes related to how motherhood was impacted by symptoms, demands of treatment, and the need to gain acceptance of living with cancer
20	Zheng et al., 2018	China	To understand the real experiences of young female breast cancer patients disclosing their condition to their children	*N* = 13, Breast cancer; married female patients under the age of 45 with at least one child	● Phenomenological research ● Semi-structured interviews ● Colaizzi analysis method	● Making decisions about disclosing disease ● The need for professional guidance ● Feeling guilty and worry about children ● Positive experiences with children
21	Park et al., 2016	USA	To describe the experience of living with advanced cancer as a parent, including illness experience, parental concerns, and treatment decision-making	*N* = 42, patients with cancer, at least 18 years old, and had at least one child younger than 18 years	● A qualitative descriptive design ● Semi-structured interviews ● Thematic content analysis	● Parental concerns about the impact of their illness and death on their children ● “Missing out” and losses of parental role and responsibilities ● Maintaining parental responsibilities despite life-limiting illness ● Parental identity influencing decision-making about treatment
22	Huang et al., 2017	China	To explore how Chinese mothers with breast cancer communicate about their illness with their children	*N* = 40, mothers with nonterminal breast cancer;receiving or having completed primary treatments (surgery, chemotherapy, radiation, etc.); having at least 1 child younger than 18 years when diagnosed	● An interpretive description study ● Individual interviews ● 3 steps coding for analysis(free coding, descriptive coding, and interpretive coding)	● Breaking the news ● Explaining to children ● Disclosing versus concealing, ● Information needs
23	Lundquist, 2017	USA	To examined the experience of fathers navigating the challenges of living with advanced cancer while parenting minor children	*N* = 11, fathers with advanced cancer diagnosis (stage III or IV) of any type, at least 3 months postdiagnosis, and actively engaged in raising at least one child under the age of 18	● Grounded theory method ● A single, in-depth, face-to-face, emistructured interview with each participant ● Not reported	● The fathers used key protective strategies to counter-balance the weight of the barriers to achieve resilience throughout the cancer experience ● Primary barriers were characterized as physical impairments, uncertainty, and financial strain ● Fathers described relying on flexibility in their roles as fathers, open communication patterns, use of supportive resources, and the ability to find meaning in their experiences as crucial to fostering resilience
24	O’Neill et al., 2018	Ireland	To explore the experiences of fathers diagnosed and living with cancer when they have parental responsibility	*N* = 10, a total of 22 interviews; fathers with parental responsibility for children(0–18 years) with a first diagnosis of cancer who were receiving treatment with curative intent and were diagnosed 2–12 months	● A hermeneutic phenomenological research design ● In-depth interviews ● Van Manen’s (1997) framework for data analysis	● Fathers’ embodied experience of the illness and its disruption to their lives ● Discovery of the significance of fatherhood and the creation of new meaning in their role ● The complexity and diversity of fathers’ roles, family structures and familial relationships
25	Rashi et al., 2015	Canada	To explore the cancer experience of parents and their perceptions of supportive strategies to assist them with illness- and family-related challenges.	*N* = 12, 5 mothers and 7 fathers with a first cancer diagnosis who received treatment at the cancer clinic within the previous year, and participants had to have at least one minor child living with them	● Qualitative, descriptive research design ● Semistructured, audio-recorded interviews ● Not reported	● Parental self-activated strategies, including maintaining child routines, selective disclosure, strength and positivity, adapting to illness-related physical changes, and connecting with others who are similar ● Tangible social networks that meet transportation, child care, meal care, and psychoemotional support needs ● Suggestions to enhance person- and family-centered care, including information to benefit the children, coordination of appointments, optimizing timing for support services, and the need for more tangible support
26	Strickland et al., 2015	Canada	To investigate how young mothers manage their maternal roles and responsibilities during their journey as patients with cancer	*N* = 18, women aged 27–45 years when diagnosed and who were concluding or had concluded treatment for breast or non-ovarian reproductive cancer, being mother at least one child aged 17 years or younger	● Glaserian grounded theory (GT) methodology ● Face-to-face semi Structured interviews ● Based on GT:building substantive codes and advancing abstraction to theoretical codes	● Customizing Exposure ● Reducing Disruption to Family Life ● Finding New Ways to Be Close ● Increasing Vigilance
27	Kissil et al., 2014	USA	This study examined how African American parents cope with the diagnosis and treatment of breast cancer	*N* = 9, parent diagnosed or treated for Stage I, II, or III breast cancer within the past 2 years and at least one child between the ages of 11 and 18, living with the parent and aware of the parent’s diagnosis	● Focus-group study ● Focused interviews to obtain information from individuals and interactions among individuals in a small group setting ● Content analysis	● Involvement in community of support ● Relationship with cancer ● Being the family emotional regulator ● Highlighting positives ● Spirituality
28	Asbury et al., 2014	UK	To investigate the experiences of mothers in telling their children of their diagnosis	*N* = 10, women who had recently had a diagnosis of breast cancer (1–4 years) and had completed adjuvant chemotherapy and/or radiotherapy at least 6 months prior to the interview;women who had children (2–24 years old) living at home at the time of diagnosis	● Not reported ● Semi-structured interviews ● A thematic analysis	Protecting children:Maintaining normality ● Information management ● Minimising own needs and reaction
29	Campbell-Enns and Woodgate, 2013	Canada	To explore the process of decision-making in mothers with cancer when they are mothering young children	*N* = 8, Women were diagnosed within the past 5 years with cancer of any type and stage and be a mother of one or more children under the age of 7 years at the time of diagnosis	● A qualitative research based in the principles of grounded theory ● Individual interviews ● Line-by-line coding and focused coding	● Core category: maintaining the mother-child bond ● Conditions influencing decision making ● Consequences of maintaining the mother-child bond: coping strategies
30	Davey et al., 2012	USA	To understand African American parents’ experiences parenting their school-age children while navigating breast cancer	*N* = 9, parent diagnosed with Stage I, II, or III breast cancer within the previous 2 years and at least one child between the ages of 11 and 18, living with the ill parent and aware of the parent’s diagnosis	● Not reported ● Focus groups ● Content analysis	● Increased desire to protect their children ● Parental concerns for children’s coping ● Openness and transparency with children ● Reliance on children for support ● Calibration of their own responses, ● Use of the illness experience as a teachable moment for children ● Reliance on others for parenting support.
31	Kim et al., 2012	Korea	To explore the impact of breast cancer on Korean mothers and their children following diagnosis	*N* = 7, women diagnosed with breast cancer (stage 0–2) at age 50 years or younger; women who lived with their school aged or adolescent child(ren) at time of diagnosis greater than 1 year since diagnosis, and no organ metastasis or recurrence	● A qualitative study ● One-time in-depth interviews ● Content analysis	● The delicate balance of being able to focus on self, which also was a conflicting factor in their relationship with children ● The continuing challenge of taking care of children, which was closely linked to supports, health condition, and cultural notions of parenting and lingering stigma ● The importance of informing children in a timely manner ● An overall shift in attitudes toward raising children as independent beings ● Relinquishing and re-envisioning the future for their children and themselves
32	Stiffler et al., 2008	USA	To examine maternal parenting during the time when a mother is diagnosed with and treated for breast cancer from the mothers’ and adolescent daughters’ perspectives	*N* = 9, 8 mothers and 1 of their adolescent daughters. The mothers were aged 37–46 years at the time of diagnosis, had stage 0–IV cancer, and had completed treatment 1–12 years earlier. Participants had one to four children ranging in age from 13–24 years at the time of the interview	● Empirical phenomenological research ● An open-ended, audiotaped interview ● Using a procedure adapted from Colaizzi (1978) analyze data	● A battle to be fought on many fronts—what is at stake if the battle is lost, ● I tried to tell her ● Standout moments in our family’s cancer journey, ● mobilizing to protect self while preserving parenting ● Voices of fear ● After treatment is over, you are not done
33	Elmberger et al., 2005	Sweden	To explore how women with a diagnosis of cancer (lymphoma) deal with moral concerns related to their responsibility as parents	*N* = 10, women with lymphoma who had children living at home	● Grounded theory ● Focus groups and individual interviews ● The constant comparative method guided by grounded theory	● Interrupted mothering; ● Facing a life-threatening illness and children’s reactions ● Striving to be a good mother ● Dealing with moral responsibility ● Coming to terms with mothering.
34	Helseth and Ulfsaet, 2005	Norway	To understand how cancer affects childcare through assessing the parenting experiences of cancer patients and their spouses with young children	*N* = 10, the families selected had children (aged 0–18 years) living at home and one of the parents had a serious cancer disease, but was not terminally ill at the time of inclusion	● Qualitative research ● In-depth interviews ● Following Kvale’s (1996) guidelines	● Striving for normality in an abnormal situation ● Focusing on the children’s well-being ● Balancing between different needs ● Openness and support
35	Billhult and Segesten, 2003	Sweden	To describe the experience of breast cancer in mothers with dependent children and explore what strategies these women use to handle their situation of illness in relation to the children	*N* = 10, women with children living at home and diagnosed with breast cancer	● A phenomenological approach ● Semistructured individual interviews ● Three steps(whole/parts/whole)for analysis	● To be a mother with breast cancer and dependent children ● Strategies used by mothers with breast cancer
36	Shands et al., 2000	USA	To describe mothers’ reported methods of interacting with the mothers’ school-age children about their breast cancer	*N* = 19, mothers with newly diagnosed breast cancer who had a child between 7and 12 years of age at the time of diagnosis	● Descriptive study ● The structural interview ● Content analysis	● Talking about the breast cancer ● Providing experience ● Doing things to help kids cope
37	Elmberger et al., 2000	Sweden	The aim in this study was to gain a deeper understanding of the interaction between women who have been treated for breast cancer and their children	*N* = 9, women with children aged 4 to 23, living at home at the time of diagnosis	● Grounded theory method ● Interviews ● Not reported	● Becoming Exhausted ● Facing It ● Finding Meaning ● Becoming Aware of the Need for Information and Support ● Looking to the Future ● Becoming Energized

**TABLE 3:** Quality appraisal of the included studies using the critical appraisal checklist for qualitative research (JBI, 2017).

No.	Included study	1	2	3	4	5	6	7	8	9	10	Overall appraisal decision
1	Romare Strandh et al., 2025	Y	Y	Y	Y	Y	N	N	Y	Y	Y	Included
2	Pholsena et al., 2024	U	Y	Y	Y	Y	N	N	Y	Y	Y	Included
3	Johannsen et al., 2023	U	Y	Y	Y	Y	Y	N	Y	Y	Y	Included
4	Román-Oyola et al., 2022	U	Y	Y	Y	Y	N	Y	Y	Y	Y	Included
5	Zeng et al., 2023	U	Y	Y	Y	Y	Y	Y	Y	Y	Y	Included
6	Tavares et al., 2022	U	Y	Y	Y	Y	N	N	Y	Y	Y	Included
7	Chin and Chen, 2023	Y	Y	Y	Y	Y	Y	Y	Y	Y	Y	Included
8	Zhu et al., 2022	U	Y	Y	Y	Y	N	Y	Y	Y	Y	Included
9	Hauskov Graungaard et al., 2023	U	Y	Y	Y	Y	N	N	Y	Y	Y	Included
10	Mazaheri et al., 2021	U	Y	Y	Y	Y	N	Y	Y	Y	Y	Included
11	Tamura et al., 2021	U	Y	Y	Y	Y	N	Y	Y	Y	Y	Included
12	Park et al., 2021	U	Y	Y	Y	Y	N	N	Y	Y	Y	Included
13	Lundquist et al., 2020	U	Y	Y	Y	Y	N	N	Y	Y	Y	Included
14	Chin et al., 2021	U	Y	Y	Y	Y	Y	Y	Y	Y	Y	Included
15	Huang et al., 2020	U	Y	Y	Y	Y	N	N	Y	Y	Y	Included
16	Steiner et al., 2019	Y	Y	Y	Y	Y	Y	Y	Y	Y	Y	Included
17	Loggers et al., 2019	Y	Y	Y	Y	Y	N	Y	Y	Y	Y	Included
18	Sinclair et al., 2019	U	Y	Y	Y	Y	N	N	Y	Y	Y	Included
19	Arida et al., 2019	U	Y	Y	Y	Y	N	Y	Y	N	Y	Included
20	Zheng et al., 2018	U	Y	Y	Y	Y	N	Y	Y	N	Y	Included
21	Park et al., 2016	U	Y	Y	Y	Y	N	Y	Y	Y	Y	Included
22	Huang et al., 2017	U	Y	Y	Y	Y	Y	N	Y	Y	Y	Included
23	Lundquist, 2017	Y	Y	Y	Y	Y	N	N	Y	Y	Y	Included
24	O’Neill et al., 2018	Y	Y	Y	Y	Y	N	Y	Y	Y	Y	Included
25	Rashi et al., 2015	U	Y	Y	Y	Y	N	N	Y	Y	Y	Included
26	Strickland et al., 2015	Y	Y	Y	Y	Y	Y	N	Y	Y	Y	Included
27	Kissil et al., 2014	U	Y	Y	Y	Y	Y	N	Y	Y	Y	Included
28	Asbury et al., 2014	U	Y	Y	Y	Y	N	N	Y	Y	Y	Included
29	Campbell-Enns and Woodgate, 2013	U	Y	Y	Y	Y	N	N	Y	Y	Y	Included
30	Davey et al., 2012	Y	Y	Y	Y	Y	N	N	Y	Y	Y	Included
31	Kim et al., 2012	U	Y	Y	Y	Y	N	N	Y	N	Y	Included
32	Stiffler et al., 2008	U	Y	Y	Y	Y	N	N	Y	Y	Y	Included
33	Elmberger et al., 2005	U	Y	Y	Y	Y	N	N	Y	Y	Y	Included
34	Helseth and Ulfsaet, 2005	Y	Y	Y	Y	Y	N	N	Y	Y	Y	Included
35	Billhult and Segesten, 2003	U	Y	Y	Y	Y	N	N	Y	Y	Y	Included
36	Shands et al., 2000	U	Y	Y	Y	Y	N	N	Y	Y	Y	Included
37	Elmberger et al., 2000	Y	Y	Y	Y	Y	N	N	Y	Y	Y	Included

1. Is there congruity between the stated philosophical perspective and the research methodology? 2. Is there congruity between the research methodology and the research question or objectives? 3. Is there congruity between the research methodology and the methods used to collect data? 4. Is there congruity between the research methodology and the representation and analysis of data? 5. Is there congruity between the research methodology and the interpretation of results? 6. Is there a statement locating the researcher culturally or theoretically? 7. Is the influence of the researcher on the research, and vice-versa, addressed? 8. Are participants, and their voices, adequately represented? 9. Is the research ethical according to current criteria or, for recent studies, and is there evidence of ethical approval by an appropriate body? 10. Do the conclusions drawn in the research report flow from the analysis, or interpretation, of the data? Y = Yes; *N* = No; U = Unclear.

### 3.3 Main findings of the meta-synthesis

**FIGURE 2:**
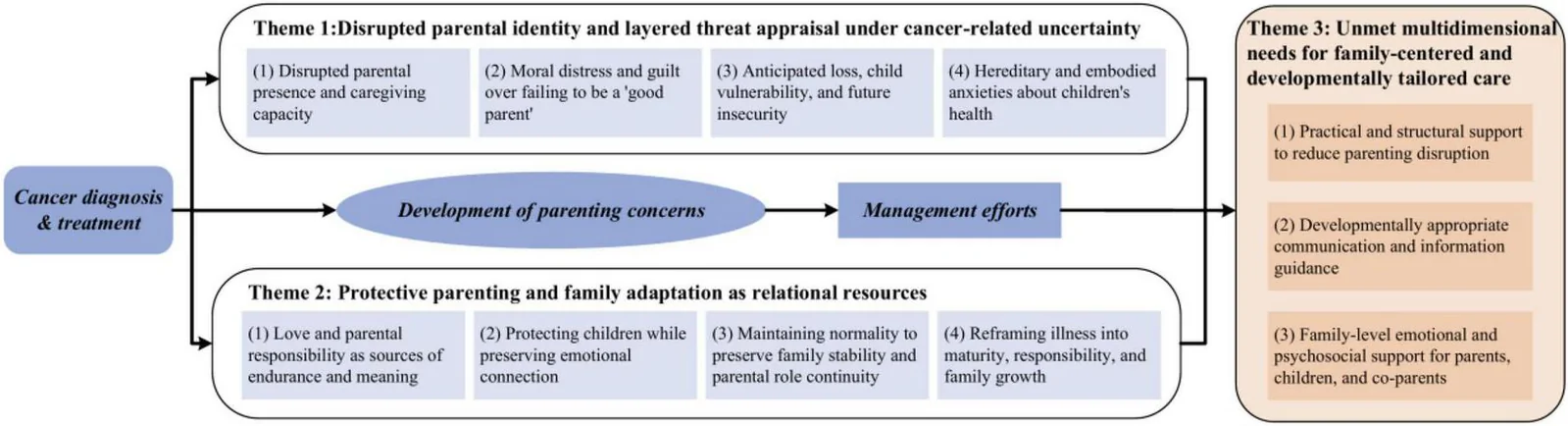
An interpretive model of parenting concern in cancer patients.

The meta-synthesis generated three themes and 11 subthemes that explain how parenting concerns develop and are managed in cancer-affected families. Across studies, cancer diagnosis and treatment undermined parents’ everyday caregiving capacity and threatened their ability to remain a “good parent”, which generated layered concerns about children’s present well-being, future security, and possible hereditary risks. At the same time, parenting concerns also activated relational resources, as parents attempted to protect children, maintain emotional connection and family normality, and reconstruct meaning through love, responsibility, and family growth. Nevertheless, these adaptive efforts were often self-initiated and insufficiently supported, highlighting the need for family-centered and developmentally tailored care. Figure 2 summarizes this interpretive model.

**Theme 1: disrupted parental identity and layered threat appraisal under cancer-related uncertainty**

This theme describes how cancer disrupted patients’ sense of themselves as parents and generated layered threat appraisals about children and the family’s future. Across the included studies, parents did not interpret cancer-related functional decline, hospitalization, and treatment burden merely as practical caregiving difficulties. Rather, these changes were understood as threats to parental presence, caregiving capacity, emotional availability, and the moral identity of being a “good parent”. As illness uncertainty persisted, threat appraisal expanded from current parenting disruption to children’s future security, possible parent-child separation, the continuity of family life, and potential hereditary risks to children’s health. Thus, parenting concern originated in the conflict between the patient role and the parental role, but more deeply reflected cancer parents’ multi-layered worries about children’s current well-being, future development, and intergenerational health risks.

1. Disrupted parental presence and caregiving capacity

The core of the parental role is to nurture, care for, accompany, guide, and emotionally support children. However, cancer imposed time, physical, and emotional constraints that created persistent conflict between being a patient and being a parent. Surgery, chemotherapy, radiotherapy, fatigue, pain, immune vulnerability, hospitalization, and medical appointments limited parents’ ability to provide daily care, supervise schoolwork, accompany children, participate in school or social activities, and maintain familiar family routines (Huang et al., 2020; Lundquist, 2017; Mazaheri et al., 2021; Rashi et al., 2015; Romare Strandh et al., 2025; Strickland et al., 2015; Tamura et al., 2021).

Several studies illustrated how the ill body directly interrupted ordinary parenting. After surgery, some mothers could not hug their children and even had to ask children to approach gently (Strickland et al., 2015). Because of swelling in the hands and declining strength, some parents could no longer drive children as before (Mazaheri et al., 2021). Caregiving disruption was particularly salient when children were young, when the patient had previously been the primary caregiver, or when the family lacked alternative support. For example, one mother with colorectal cancer was unable to care for her infant during cancer-related pain crises and had to rely on her adolescent son to prepare formula and feed his younger sibling at night (Rashi et al., 2015).

Importantly, changes in parental presence were not always experienced in a single negative direction. Studies of fathers with cancer showed that illness could also prompt some men to shift their life focus from work toward family. When their physical condition improved, some fathers participated more in housework and parent-child companionship and viewed this renewed family involvement as an opportunity to re-adapt to fatherhood (Lundquist, 2017). This finding suggests that cancer has a complex influence on parental roles: it disrupts prior caregiving capacity and family order, but may also prompt some patients to reinterpret and re-practice parenthood (Zhao et al., 2023).

2. Moral distress and guilt over failing to be a “good parent”

When cancer prevented parents from being responsible, emotionally available, and protective in the way they expected of themselves, many experienced guilt, shame, self-blame, and moral distress. Some parents described themselves as inadequate or as having failed their children because illness prevented them from providing companionship, patience, financial security, emotional availability, or continuous care (Huang et al., 2020; Mazaheri et al., 2021; Park et al., 2016; Rashi et al., 2015; Romare Strandh et al., 2025). This moral distress was intensified by the asymmetry between parents’ intentions and capacities. Parents still wanted to protect children, maintain family stability, and provide a normal childhood, but their bodies, treatment demands, and emotional burden made this difficult.

Some children showed separation anxiety, fear, silence, or behavioral changes after parents left home for treatment or were absent for extended periods. Parents often interpreted these changes as evidence that they had not sufficiently protected or accompanied their children, which deepened their self-blame and helplessness (Elmberger et al., 2000; Román-Oyola et al., 2022). At the same time, one study reported that some parents sought a better caregiving environment or even foster-parent arrangements for children, which partly relieved their guilt and helped them preserve a sense of moral responsibility in parenting (O’Neill et al., 2018).

This internal conflict around being a “good parent” was shaped by gender. Mothers often worried about being irreplaceable in daily care and emotional caregiving, whereas fathers sometimes struggled with the loss of provider identity and with cultural expectations that men should remain strong, independent, and self-sufficient (Huang et al., 2020; Lundquist, 2017).

3. Anticipated loss, child vulnerability, and future insecurity

This subtheme concerns parents’ future-oriented threat appraisal. Cancer is a severe illness associated with recurrence, progression, and death. Parents worried that their children might not receive adequate care, emotional protection, financial resources, or parental guidance in the future (Elmberger et al., 2000; Huang et al., 2020; Lundquist et al., 2020; Park et al., 2016; Romare Strandh et al., 2025; Tamura et al., 2021; Zheng et al., 2018). These worries were especially strong among patients with advanced disease, single parents, parents of young children, and those who believed that their parental role could not be replaced (Huang et al., 2020; Lundquist et al., 2020; Park et al., 2016; Romare Strandh et al., 2025). One participant described the fear of leaving a young child without a mother, emphasizing that a child who loses a mother can never truly find another one (Huang et al., 2020).

Parents’ concerns about children’s vulnerability involved both immediate emotional reactions and long-term developmental consequences. Studies reported that children, after learning about parental illness, could show anxiety, withdrawal, sadness, anger, academic difficulties, somatic complaints, behavioral changes, or excessive responsibility-taking (Chin and Chen, 2023; Elmberger et al., 2000; Hauskov Graungaard et al., 2023; Lundquist et al., 2020; Mazaheri et al., 2021; Pholsena et al., 2024; Zheng et al., 2018; Zhu et al., 2022). Parents often struggled to distinguish whether children’s emotional and behavioral changes reflected ordinary adolescence or distress caused by parental cancer. This uncertainty increased parents’ helplessness and intensified their need for professional guidance. Therefore, anticipated loss was not limited to fear of death; it also involved fear that cancer would disturb children’s trust, development, emotional security, and future family structure (Kim et al., 2012; Pholsena et al., 2024).

4. Hereditary and embodied anxieties about children’s health

Some cancers have clear or perceived hereditary tendencies. In studies involving breast cancer and other cancers with known or suspected familial risk, parents worried that children might inherit susceptibility to cancer or later encounter similar illness trajectories (Davey et al., 2012; Huang et al., 2017; Steiner et al., 2019; Zhu et al., 2022). This concern linked the parent’s diseased body to the child’s imagined future body. Some parents experienced guilt because cancer seemed to threaten children not only emotionally but also biologically.

Hereditary anxiety was closely tied to information needs. Parents wanted accurate and individualized guidance about genetic risk, screening, prevention, and how to discuss risk with children without creating unnecessary fear (Davey et al., 2012; Huang et al., 2017; Steiner et al., 2019). For parents of daughters, particularly in breast cancer contexts, this concern sometimes shaped health education, monitoring behaviors, and communication about future screening (Davey et al., 2012; Zhu et al., 2022). The synthesis indicates that hereditary anxiety expands the conceptual scope of parenting concerns: children are not only vulnerable family members who may be affected by parental illness, but also imagined future patients whose health risks may be connected to the parent’s diagnosis.

**Theme 2: protective parenting and family adaptation as relational resources**

This theme reveals the protective parenting practices and family adaptation processes mobilized by cancer parents after identity disruption and threat appraisal. Parenting concern was not expressed solely as distress, guilt, or helplessness; it could also activate relational resources within parents and families. The included studies showed that children and parental love often became important sources of treatment persistence, meaning reconstruction, and adaptation to the new identity of being a parent with cancer. Parents also constantly balanced child protection and emotional connection, using selective communication, family routines, and ordinary daily life to reduce the impact of illness on children’s lives and psychological state. Meanwhile, some families demonstrated increased responsibility, maturity, gratitude, and cohesion while facing illness together.

1. Love and parental responsibility as sources of endurance and meaning

Across the included studies, children were frequently described as a central reason for cancer parents to continue treatment, endure suffering, and remain engaged in life (Arida et al., 2019; Elmberger et al., 2000; Lundquist et al., 2020; Román-Oyola et al., 2022; Romare Strandh et al., 2025; Tamura et al., 2021; Zheng et al., 2018). Parents drew strength from love, responsibility, and the desire to witness their children’s growth. One participant stated, ‘I felt I was obligated to recover again. I had to fight against my own suffering’ (Elmberger et al., 2000). For many mothers, strong attachment to children and maternal responsibility compelled them to endure the suffering caused by disease. Conversely, children also provided female patients with security, motivation to continue treatment, and energy for daily living, thereby reducing the psychological consumption caused by cancer-related stress and adverse events (Román-Oyola et al., 2022; Romare Strandh et al., 2025).

Thus, parenting concern contained a paradox: the same bond that intensified fear and guilt also provided meaning, motivation, and emotional energy. Contact with children, photographs, telephone calls, play, daily caregiving, and shared small moments could all serve as emotional replenishment (Arida et al., 2019; Román-Oyola et al., 2022). These experiences helped parents reconstruct meaning during illness and resist being defined only as patients.

2. Protecting children while preserving emotional connection

Parents used multiple protective strategies to reduce children’s distress while trying to preserve intimacy. Some concealed information, delayed disclosure, softened explanations, or avoided showing bodily vulnerability; others chose open communication, honest preparation, or shared discussion about uncertainty and death (Asbury et al., 2014; Elmberger et al., 2000, 2005; Park et al., 2021; Shands et al., 2000; Tamura et al., 2021; Zeng et al., 2023). These strategies varied according to children’s age, maturity, family communication patterns, disease stage, and cultural expectations about protecting children from suffering.

For example, one father explained that he did not want his children to see him in pain and therefore invited them to the hospital only when he felt relatively well, because he wanted to protect the image of fatherhood (Tamura et al., 2021). These apparently opposite strategies can both be understood as forms of protective parenting. Parents who concealed information often aimed to prevent fear, preserve childhood normality, or maintain an image of parental strength (Huang et al., 2020; Kim et al., 2012). Parents who disclosed information often aimed to maintain trust, reduce confusion, and prepare children for change (Hauskov Graungaard et al., 2023; Shands et al., 2000). However, both groups faced the same dilemma: cancer parents often did not know how to manage cancer-related information that might overwhelm children while still protecting them. Many therefore hoped for professional guidance regarding timing, wording, and developmentally appropriate communication (Huang et al., 2017, 2020; Zheng et al., 2018; Zhu et al., 2022).

3. Maintaining normality to preserve family stability and parental role continuity

Another protective strategy was the attempt to maintain ordinary family life. Parents tried to preserve routines, keep children engaged in school and leisure activities, spend time with children when physically able, and adapt activities according to changing energy levels (Helseth and Ulfsaet, 2005; Lundquist, 2017; Román-Oyola et al., 2022; Romare Strandh et al., 2025; Shands et al., 2000; Tamura et al., 2021). Maintaining normality helped parents preserve continuity in the parental role and reassured children that family life had not been completely replaced by illness.

Behind the effort to maintain normality was also a process in which cancer patients reconsidered their parental role and actively adjusted to the new identity of being a parent with cancer. This difficult and challenging experience sometimes promoted positive changes and helped patients become more reflective parents. For example, some parents shifted from physically demanding activities to quieter interactions, relied on partners or relatives for practical tasks, and focused on emotionally meaningful moments (Shands et al., 2000). For fathers in some studies, illness created unexpected opportunities to spend more time at home and renegotiate the fathering role (Lundquist, 2017; O’Neill et al., 2018; Tamura et al., 2021). One participant stated, ‘I think the cancer made me into a father. Until then (before becoming ill), I really didn’t have that sort of awareness, you know?’ (Tamura et al., 2021).

4. Reframing illness into maturity, responsibility, and family growth

Some parents and families reframed the cancer experience as an opportunity for learning, responsibility, gratitude, and family growth (Davey et al., 2012; Elmberger et al., 2000; Kim et al., 2012; Lundquist et al., 2020; Park et al., 2016; Tamura et al., 2021; Zheng et al., 2018; Zeng et al., 2023). By explaining disease causes, treatment processes, and health risks to children, patients guided children to pay attention to health, develop healthier lifestyles, and learn how to face crisis and uncertainty. In families affected by breast cancer or other cancers with hereditary risk, parents were especially likely to transform their illness experience into health reminders and life education for children. One mother reported telling her son why she became ill, encouraging him to exercise more, remain happy, and avoid unhealthy habits (Huang et al., 2017).

Cancer parents also hoped to help children learn how to face adversity, take responsibility, and live independently. Some parents, worried that they might not be able to accompany children in the future, intentionally strengthened children’s life skills, values, and psychological resilience and encouraged siblings to support one another so that they could cope better with future uncertainty (Davey et al., 2012; Elmberger et al., 2000; Park et al., 2016). Children also participated in this adaptation process. Several studies showed that after learning about parental illness, some children actively took on household tasks, cared for siblings, accompanied ill parents, searched for disease-related information, and participated in parents’ rehabilitation (Huang et al., 2020; Kim et al., 2012; Lundquist et al., 2020; Zheng et al., 2018; Zeng et al., 2023). These behaviors reassured parents and were interpreted as signs of increased maturity and responsibility (Lundquist et al., 2020; Zheng et al., 2018; Zeng et al., 2023).

At the family level, cancer also prompted some families to re-examine relationships and life priorities. After illness, patients reported paying more attention to family members’ feelings, shared responsibilities, and time spent together, which strengthened family interaction and cohesion (Helseth and Ulfsaet, 2005; Mazaheri et al., 2021; Tamura et al., 2021). Some patients described valuing small moments of happiness, such as children’s smiles, hugs, and bedtime companionship, more than before (Lundquist, 2017; O’Neill et al., 2018; Romare Strandh et al., 2025; Tamura et al., 2021).

**Theme 3: unmet multidimensional needs for family-centered and developmentally tailored care**

This theme reflects the support needs that remained insufficiently met when cancer parents coped with parenting concerns. The included studies showed that cancer parents needed more than general emotional support. They required comprehensive care that was family-centered and adapted to children’s developmental stages and illness contexts, including practical and structural support to reduce parenting disruption, communication guidance appropriate to children’s age and understanding, and psychosocial support for parents, children, and co-parents.

1. Practical and structural support to reduce parenting disruption

To cope with parenting concerns, cancer parents often considered seeking professional support and expressed specific expectations about the content and form of such assistance. Across studies, parents repeatedly mentioned practical needs such as transportation, childcare, meal preparation, household assistance, and financial support (Kim et al., 2012; Lundquist, 2017; Rashi et al., 2015; Romare Strandh et al., 2025; Steiner et al., 2019). These practical resources directly affected whether parents could accompany children, maintain family routines, and reduce guilt over interrupted caregiving.

More importantly, structural barriers within healthcare systems could intensify parenting concerns. Long waiting times, poorly coordinated appointments, limited family-oriented services, and insufficient referral pathways placed additional burdens on parents who were already managing both treatment and childcare (Lundquist, 2017). Conversely, practical support from partners, grandparents, friends, neighbors, community organizations, schools, and hospital social workers helped buffer parenting disruption (Rashi et al., 2015; Romare Strandh et al., 2025).

2. Developmentally appropriate communication and information guidance

Multiple studies showed that cancer parents commonly hoped to receive systematic information support from professional institutions or healthcare providers. Their needs included not only medical information about treatment, side effects, prognosis, hereditary risk, and health monitoring, but also guidance on how to explain cancer, treatment processes, bodily changes, recurrence risk, and even death to children (Asbury et al., 2014; Park et al., 2021; Rashi et al., 2015; Sinclair et al., 2019; Steiner et al., 2019; Zeng et al., 2023). For cancer parents, communicating about cancer with children required adjustment according to children’s age, cognitive level, emotional capacity, and family context. Parents expected healthcare professionals to provide developmentally appropriate communication guidance and help them decide when to talk, how much to say, how to say it, and how to respond to children’s questions and emotional reactions.

Parents also had high expectations regarding the quality and timing of information support. Generic or poorly matched materials could fail to help family communication and might even increase children’s misunderstanding or fear (Rashi et al., 2015; Steiner et al., 2019). For example, one participant noted that a book provided by healthcare professionals left the child with a strong impression of hair loss, even though not all patients experience this side effect (Rashi et al., 2015). Parents therefore wanted diverse, accessible professional resources, including educational materials on cancer and parent-child communication, information sessions, counseling services, and support groups (Rashi et al., 2015).

In addition, cancer parents needed individualized professional guidance that respected family background. Some fathers described tension between needing support and seeking help because help-seeking could threaten masculine ideals of independence (Lundquist, 2017). Some patients facing hereditary risk also hoped for concrete advice on children’s future screening and health management. As one participant emphasized, healthcare providers need to listen to each patient because what each parent and family needs is individual, and a generalized cancer support program may not fit a particular family (Rashi et al., 2015).

3. Family-level emotional and psychosocial support for parents, children, and co-parents

Parents’ needs extended to the family system. Patients wanted psychosocial support for their own distress, for children’s emotional and behavioral reactions, and for partners or co-parents who were taking on additional caregiving responsibilities (Chin and Chen, 2023; Elmberger et al., 2000; Hauskov Graungaard et al., 2023; Johannsen et al., 2023; Kim et al., 2012; Pholsena et al., 2024; Tavares et al., 2022). Several studies indicated that children might experience fear, loneliness, anger, school problems, somatic symptoms, or confusion, while parents often felt uncertain about how to interpret and respond to these reactions and strongly hoped for professional help (Kim et al., 2012; Pholsena et al., 2024). One parent described being lost and distressed because she did not know how to help her daughter when the child’s personality changed as the parent’s illness worsened (Pholsena et al., 2024).

For parents with advanced cancer, family-level psychosocial support also pointed toward future-oriented preparation. Although some cancer parents had already discussed death with children, arranged future caregivers, or strengthened children’s life skills (Chin and Chen, 2023; Elmberger et al., 2000; Huang et al., 2017; Zheng et al., 2018), these efforts relied heavily on parents’ individual experience and lacked professional guidance.

## 4 Discussion

This study integrated 37 qualitative studies on parenting concerns among cancer patients and developed an interpretive understanding of this phenomenon. Rather than treating parenting concern only as psychological distress or unmet need, our synthesis suggests that it is an embodied, relational, and family-adaptive process organized around the maintenance of ‘good parent’ identity, child protection, and family continuity under cancer-related uncertainty. Cancer diagnosis and treatment not only weaken daily caregiving capacity, but also threaten the continuity and moral meaning of parental identity. This leads parents to conduct layered threat appraisals concerning children’s present well-being, future security, family continuity, and potential hereditary risk. At the same time, patients are not merely passive sufferers; they mobilize relational resources through love and responsibility, protective communication, maintenance of family normality, and meaning reconstruction. However, these adaptive efforts are often self-initiated and insufficiently supported, indicating that oncology care should more explicitly incorporate family-centered and developmentally tailored support (Ellis et al., 2017; Inhestern et al., 2016c; Kuo et al., 2012; Semple and McCance, 2010).

### 4.1 Understanding parenting concern through role conflict and layered threat appraisal

One important finding of this study is that parenting concern among cancer patients is not only a conflict between the roles of ‘patient’ and ‘parent,’ but also a layered threat appraisal of parental identity, child safety, and the family’s future under cancer-related uncertainty. Previous studies have emphasized the multiple burdens faced by cancer parents in relation to treatment, physical decline, childcare, and family care (Li et al., 2024; Zhang et al., 2025). Our synthesis further shows that what truly aggravates distress is not simply caregiving disruption, but the meaning parents attach to these disruptions: losing parental authority, becoming a ‘bad parent,’ causing emotional harm, experiencing guilt, shame, and moral distress, witnessing children’s emotional instability or maladaptive behavior, and fearing that children will be unprepared for the future.

These findings extend earlier work on parenting concerns (Inhestern et al., 2021; Jewett et al., 2020; Li et al., 2024; Mori et al., 2023; Zhang et al., 2025; Zhao et al., 2024) and are consistent with role identity theory, which suggests that identity-related distress arises when individuals cannot enact roles that are central to their self-definition (Stryker and Burke, 2000). This perspective helps explain why parenting concern may persist even when other family members temporarily assume practical caregiving tasks: the issue is not only whether childcare is completed, but whether the patient can still experience continuity, legitimacy, and moral worth in the parental role.

The meaning of this role conflict is also culturally and gendered. In East Asian cultural contexts, family responsibility, parental sacrifice, children’s education, and protection of children from suffering may intensify the moral pressure to remain a competent and self-sacrificing parent (Hahne et al., 2020; Li et al., 2026). In some Western studies, open communication, children’s right to know, individual autonomy, and professional psychosocial support were more strongly emphasized (Hailey et al., 2018; van der Zwaag et al., 2026). These differences should not be treated as a simple opposition between concealment and openness; rather, they reflect different moral routes through which parents attempt to protect children while maintaining trust. Similarly, mothers’ concerns were often linked to daily care, emotional availability, body image, and perceived irreplaceability, whereas fathers’ concerns more often involved provider identity, masculine ideals of strength, independence, and difficulty seeking help (Huang et al., 2020; Lundquist, 2017).

In addition, this study incorporates hereditary and embodied anxiety into the conceptual scope of parenting concern. In cancers with clear or perceived hereditary risk, such as breast cancer, parental worry is directed not only toward children’s current psychological adjustment but also toward their possible future bodily risk. Therefore, future assessment of parenting concern in cancer patients should not focus only on parent-child communication and caregiving difficulty; it should also include genetic risk, health monitoring, and parents’ guilt and uncertainty about children’s future physical health.

### 4.2 Mitigating parenting concern through relational resources and family adaptation

This study found that parenting concern is not solely a negative burden; in some families, it also activates protective parenting and family adaptation. At the parental level, love and responsibility toward children often become important sources of treatment persistence, suffering endurance, and life engagement. Parents also reduce the impact of illness on children by using selective communication, maintaining family routines, adjusting parenting activities, and reconstructing life meaning.

At the child level, some children learn necessary life skills earlier, become more independent, or participate more actively in family responsibilities. These positive changes reported by parents and children are consistent with posttraumatic growth theory, which describes positive psychological transformation after highly challenging life events (Tedeschi and Calhoun, 1996). At the family level, some families facing cancer demonstrated stronger responsibility, family cohesion, attention to children’s growth, and appreciation of everyday life. This finding is consistent with family resilience theory, which proposes that families maintain or restore functioning in major adversity through shared meaning-making, role reorganization, emotional connection, and collaborative coping (Walsh, 2002).

Importantly, this study does not imply that cancer itself is positive, nor does it romanticize the growth of patients and families. The so-called ‘positive forces’ are not automatic consequences of illness, but relational resources mobilized by family members through love, responsibility, values, and mutual support amid suffering and uncertainty. This understanding helps avoid pathologizing parenting concern as only anxiety or burden. It also suggests that interventions should not only reduce anxiety and guilt, but also help families identify existing strengths, maintain meaningful parent-child interaction, promote age-appropriate communication, and support shared coping.

Cultural and family-structure sensitivity should be embedded in this adaptive interpretation. Families differ in how they define protection, openness, parental authority, and children’s participation in illness-related communication. Clinical support should therefore not impose a single communication standard, but should respect family values, cultural background, children’s developmental stage, and the family’s prior communication patterns. This approach is consistent with cultural competence frameworks in healthcare, which emphasize adapting care to the cultural context of patients, families, and communities (Campinha-Bacote, 2002).

### 4.3 Addressing unmet parenting needs through multidimensional and family-centered support

The findings of this study suggest that parenting concerns should be incorporated into routine assessment in oncology care. Clinical assessment should not be limited to asking whether patients have children. It should further explore children’s ages, caregiving arrangements, difficulties in parent-child communication, children’s emotional and behavioral reactions, economic and practical support, future caregiving plans, and concerns about hereditary risk. Particular attention should be given to patients with advanced cancer, single parents, families with young children, families with insufficient social support, and families facing possible hereditary cancer risk.

Second, healthcare professionals should provide cancer parents with communication support that is adapted to children’s developmental stage. Clinical teams can develop age-specific communication scripts, picture books, visual materials, question prompt lists, and family conversation tools. These resources can help parents communicate with children in a gradual, accurate, and understandable way (Shands et al., 2000), while avoiding information overload, vague explanations, or poorly matched materials that may generate additional fear (Sievers et al., 2024).

Third, the target of support should expand from the cancer parent to the family system. Children may experience fear, withdrawal, anger, school difficulties, somatic discomfort, or premature caregiving responsibility. Spouses, grandparents, and other co-parents may also experience stress because they assume additional parenting and caregiving tasks. Oncology care should therefore establish family-level psychosocial support pathways, including referral to child psychology, social work, school counseling, or community cancer-support services when necessary. Families facing hereditary cancer concerns should receive timely genetic counseling or referral so that parents’ guilt and uncertainty can be addressed with accurate information.

Fourth, practical and structural support should be regarded as an important component of psychosocial care. Transportation, childcare, meal support, flexible appointment scheduling, financial counseling, respite care, and coordination with schools and community organizations may directly reduce parenting disruption and patient guilt (Rashi et al., 2015; Romare Strandh et al., 2025). For single parents, parents with advanced disease, and families with limited social resources, such concrete assistance should be integrated into family-centered care pathways rather than treated as supplementary support.

Finally, for parents with advanced cancer, care should also include future-oriented preparation. Some parents need help enabling children to understand disease progression, possible parent-child separation, and death, while also wishing to maintain emotional connection through memory-making, letters, videos, keepsakes, or the transmission of life values (Park et al., 2016; Taneja et al., 2024). This type of support should be offered sensitively according to disease stage, parental readiness, cultural background, and children’s developmental level. The aim is to avoid placing a premature psychological burden on children while also preventing families from losing opportunities for preparation because death-related topics are avoided.

### 4.4 Limitations

Several limitations should be considered. First, many included qualitative studies did not adequately describe their philosophical paradigms, researchers’ cultural or theoretical positions, or the influence of researchers on data collection and interpretation. This incomplete reporting of reflexivity may have reduced transparency in part of the evidence base and should be considered when interpreting the synthesized findings. Second, although the search included both Chinese and English databases, only studies published in Chinese or English were included. Relevant studies published in other languages may therefore have been missed, and language bias cannot be excluded. Third, cancer types, disease stages, children’s ages, family structures, and cultural contexts varied substantially across included studies. This diversity strengthened conceptual breadth but limited the ability to specify subgroup mechanisms. Finally, because parenting concerns inherently involve bidirectional dynamics between cancer patients and their children, future research should adopt a dual-perspective approach by concurrently investigating parents’ subjective caregiving experiences and children’s perceptions of parental emotional states. Particular attention should be paid to fathers, single parents, co-parents, hereditary cancer contexts, advanced cancer, and families from underrepresented cultural groups.

### 4.5 Clinical implications

Parenting concerns should be treated as a routine, family-level psychosocial issue in oncology care. Healthcare professionals can operationalize the conceptual model from this synthesis to direct actionable interventions: mitigating practical caregiving disruptions through flexible scheduling, reducing threat appraisals via targeted genetic and prognostic counseling, and equipping parents with tangible, age-specific conversation toolkits rather than generic communication advice. Furthermore, structured clinical assessments and multidisciplinary referral pathways must be culturally adapted and flexible enough to proactively address gender-specific distress, single-parent families, hereditary cancer risks, and differences in children’s developmental stages.

## 5 Conclusion

This qualitative meta-synthesis shows that parenting concern among cancer patients is a multidimensional and relational process shaped by cancer-related uncertainty. It involves disrupted parental presence and caregiving capacity, moral distress over failing to be a ‘good parent,’ anticipated loss and future insecurity, and hereditary anxieties about children’s health. At the same time, parenting concern can mobilize protective parenting and family adaptation through love, parental responsibility, emotional connection, maintenance of normality, and the reframing of illness into maturity, responsibility, and family growth.

The findings indicate that cancer parents’ support needs are multidimensional and often unmet. Effective care should integrate routine assessment of parenting concerns, developmentally appropriate communication guidance, practical and structural support, family-level psychosocial referral, genetic counseling when relevant, and future-oriented preparation for families facing advanced illness. Particular attention should be given to patients with dependent children who face advanced or hereditary cancer, single parenthood, limited family resources, young or highly distressed children, or insufficient co-parenting support. By recognizing both the burden and adaptive potential embedded in parenting concern, healthcare professionals can better support cancer patients in preserving parental identity, protecting children, and maintaining family continuity during cancer care.

## Data Availability

The original contributions presented in the study are included in the article/Supplementary material, further inquiries can be directed to the corresponding author.
