# Supplementary material for: Parenting concerns among patients with cancer: a qualitative meta-synthesis
Source: Front Psychol. 2026 Sep 3;17:1801763. doi: 10.3389/fpsyg.2026.1801763 (PMC13582470; doi:10.3389/fpsyg.2026.1801763)
Supplement: Supplementary file 1 [file Table_1.docx]

**Search strategy used in the electronic databases**

| **1. PubMed** | | |
| --- | --- | --- |
| **Number** | **Search strategy** | **Searching results** |
| #1 | "Neoplasms"[MeSH Terms] OR "Carcinoma"[MeSH Terms] OR "Sarcoma"[MeSH Terms] OR "Cancer"[Title/Abstract] OR "Neoplasms"[Title/Abstract] OR "Carcinoma"[Title/Abstract] OR "Sarcoma"[Title/Abstract] OR "Tumor"[Title/Abstract] OR "malignan*"[Title/Abstract] OR "malignant neoplasms"[Title/Abstract] | 5307344 |
| #2 | "Parenting"[MeSH Terms] OR "parenting concern"[Title/Abstract] OR "parenting stress"[Title/Abstract] OR "parenting risk"[Title/Abstract] OR "parenting worries"[Title/Abstract] OR "Nurture"[Title/Abstract] OR "Nourish"[Title/Abstract] OR "Raising"[Title/Abstract] OR "Breeding"[Title/Abstract] OR "bring up"[Title/Abstract] OR "Rear"[Title/Abstract] | 214540 |
| #3 | "qualitative research"[MeSH Terms] OR "phenomeno*"[Title/Abstract] OR "ethnogra*"[Title/Abstract] OR "ethnolog*"[Title/Abstract] OR "field study"[Title/Abstract] OR "grounded theory"[Title/Abstract] OR "focus group"[Title/Abstract] OR "narrative analysis"[Title/Abstract] OR "Interview"[Title/Abstract] OR "action research"[Title/Abstract] OR "participant observ*"[Title/Abstract] | 631979 |
| #4 | #1 AND #2 AND #3 | 327 |
| **2. Web of Science** | | |
| **Number** | **Search strategy** | **Searching results** |
| #1 | (((((((TI=(Cancer)) OR TI=(Neoplasms)) OR TI=(Carcinoma)) OR TI=(Sarcoma )) OR TI=(Tumor)) OR TI=(Malignan* )) OR TI=(malignant neoplasms)) | 3653151 |
| #2 | ((((((((((TI=(Parenting )) OR TI=(Parenting concern)) OR TI=(Parenting stress)) OR TI=(Parenting risk)) OR TI=(Parenting worries )) OR TI=(Nurture)) OR TI=(Nourish)) OR TI=(Raising)) OR TI=(Breeding)) OR TI=(Bring up)) OR TI=(Rear ) | 221188 |
| #3 | ((((((((((TI=(Qualitative research)) OR TI=(Phenomeno*)) OR TI=(Ethnogra*)) OR TI=(Ethnolog*)) OR TI=(Field study)) OR TI=(Grounded theory )) OR TI=(Focus group )) OR TI=(Narrative analysis)) OR TI=(Interview)) OR TI=(Action research)) OR TI=(Participant observ* ) | 234783 |
| #4 | #1 AND #2 AND #3 | 9 |
| **3. Cochrane library** | | |
| **Number** | **Search strategy** | **Searching results** |
| #1 | MeSH descriptor: [Neoplasms] explode all trees | 128820 |
| #2 | MeSH descriptor: [Carcinoma] explode all trees | 20751 |
| #3 | MeSH descriptor: [Sarcoma] explode all trees | 1606 |
| #4 | #1 OR #2 OR #3 | 128820 |
| #5 | ((Cancer OR Neoplasms OR Carcinoma OR Sarcoma OR Tumor OR Malignan* OR malignant neoplasms)):ti,ab,kw | 284970 |
| #6 | #4 OR #5 | 298653 |
| #7 | MeSH descriptor: [Parenting] explode all trees | 2215 |
| #8 | ((Parenting concern OR Parenting stress OR Parenting risk OR Parenting worries OR Nurture OR Nourish OR Raising OR Breeding OR Bring up OR Rear)):ti,ab,kw | 9751 |
| #9 | #7 OR #8 | 10856 |
| #10 | MeSH descriptor: [Qualitative Research] explode all trees | 2510 |
| #11 | MeSH descriptor: [Grounded Theory] explode all trees | 27 |
| #12 | MeSH descriptor: [Focus Groups] explode all trees | 1121 |
| #13 | #10 OR #11 OR #12 | 3344 |
| #14 | ((Phenomeno* OR Ethnogra* OR Ethnolog* OR Field study OR Narrative analysis OR Interview OR Action research OR Participant observ*)):ti,ab,kw | 83275 |
| #15 | #13 OR #14 | 85143 |
| #16 | #6 AND #9 AND #15 | 118 |
| **4. Embase** | | |
| **Number** | **Search strategy** | **Searching results** |
| #1 | 'neoplasm'/exp OR 'carcinoma'/exp OR 'sarcoma'/exp OR 'malignant neoplasm'/exp | 6629972 |
| #2 | 'cancer':ab,ti OR 'neoplasms':ab,ti OR 'carcinoma':ab,ti OR 'sarcoma':ab,ti OR 'tumor':ab,ti OR 'malignan*':ab,ti OR 'malignant neoplasms':ab,ti | 5420973 |
| #3 | 'parenting':ab,ti OR 'parenting concern':ab,ti OR 'parenting stress':ab,ti OR 'parenting risk':ab,ti OR 'parenting worries':ab,ti OR 'nurture':ab,ti OR 'nourish':ab,ti OR 'raising':ab,ti OR 'breeding':ab,ti OR 'bring up':ab,ti OR 'rear':ab,ti | 235963 |
| #4 | 'qualitative research'/exp OR 'grounded theory'/exp OR 'focus group'/exp OR 'narrative analysis'/exp OR 'interview'/exp OR 'action research'/exp OR 'participant observation'/exp OR 'phenomenology'/exp OR 'ethnographic research'/exp OR 'field study'/exp | 528426 |
| #5 | 'qualitative research':ab,ti OR 'phenomeno*':ab,ti OR 'ethnogra*':ab,ti OR 'ethnolog*':ab,ti OR 'field study':ab,ti OR 'grounded theory':ab,ti OR 'focus group':ab,ti OR 'narrative analysis':ab,ti OR 'interview':ab,ti OR 'action research':ab,ti OR 'participant observ*':ab,ti | 711369 |
| #6 | #1 AND #2 | 7552942 |
| #7 | #4 AND #5 | 1017417 |
| #8 | #3 AND #6 AND #7 | 823 |
| **5. CINAHL** | | |
| **Number** | **Search strategy** | **Searching results** |
| S1 | (MH "Neoplasms") OR (MH "Carcinoma") OR (MH "Sarcoma") | 109103 |
| S2 | (TI cancer OR TI neoplasms OR TI carcinoma OR TI sarcoma OR TI tumor OR TI Malignan* OR TI malignant neoplasm) | 525558 |
| S3 | S1 OR S2 | 565124 |
| S4 | (MH "Parenting") | 21848 |
| S5 | TI Parenting OR TI Parenting concern OR TI Parenting stress OR TI Parenting worries OR TI Nurture OR TI Nourish OR TI Raising OR TI Breeding OR TI Bring up OR TI Rear | 14320 |
| S6 | S4 OR S5 | 29482 |
| S7 | (MH "Qualitative Studies") OR (MH "Focus Groups") OR (MH "Grounded Theory") OR (MH "Interviews+") | 344812 |
| S8 | TI qualitative research OR TI Phenomeno* OR TI Ethnogra* OR TI Ethnolog* OR TI field study OR TI grounded theory OR TI focus group OR TI narrative analysis OR TI interview OR TI action research OR TI Participant observ* | 41538 |
| S9 | S7 OR S8 | 367067 |
| S10 | S3 AND S6 AND S9 | 71 |
| **6. EBSCO** | | |
| **Number** | **Search strategy** | **Searching results** |
| S1 | TI cancer OR TI neoplasms OR TI carcinoma OR TI sarcoma OR TI tumor OR TI Malignan* OR TI malignant neoplasm | 3653030 |
| S2 | TI parenting OR TI parenting concern OR TI parenting stress OR TI Parenting risk OR TI Parenting worries OR TI Nurture OR TI Nourish OR TI raising OR TI Breeding OR TI Bring up OR TI Rear | 164443 |
| S3 | TI qualitative research OR TI Phenomeno* OR TI Ethnogra* OR TI Ethnolog* OR TI field study OR TI grounded theory OR TI focus group OR TI narrative analysis OR TI interview OR TI action research OR TI Participant observ* | 394333 |
| S4 | S1 AND S2 AND S3 | 4 |
| **7. ProQuest** | | |
| **Number** | **Search strategy** | **Searching results** |
| S1 | title(Cancer) OR title(Neoplasms ) OR title(Carcinoma ) OR title(Sarcoma ) OR title(tumor ) OR title( Malignan* ) OR title(malignant neoplasms) | 405699 |
| S2 | title(Parenting ) OR title(Parenting concern ) OR title(Parenting stress ) OR title(Parenting risk) OR title(Parenting worries) OR title(Nurture) OR title(Nourish) OR title(Raising) OR title(Breeding) OR title(Bring up) OR title(rear) | 38269 |
| S3 | title(Qualitative research) OR title(Phenomeno*) OR title(Ethnogra*) OR title(Ethnolog*) OR title(Field study) OR title(Grounded theory) OR title(Focus group) OR title(Narrative analysis) OR title(Interview) OR title(Action research) OR title(Participant observ*) | 75052 |
| S4 | S1 AND S2 AND S3 | 1 |
| **8. China National Knowledge Infrastructure（中国知网）** | | |
| **Number** | **Search strategy** | **Searching results** |
| #1 | TKA=（‘肿瘤’+‘癌症’+‘癌’+‘恶性肿瘤’+‘瘤’） AND TKA=（‘养育忧虑’+‘养育’+‘抚养’+‘忧虑’+‘压力’+‘感受’+‘体验’） AND TKA=（‘质性研究’+‘质性访谈’+‘定性研究’+‘质性’+‘定性’+‘现象学’+‘扎根理论’+‘描述性分析’） | 1640 |
| **9. Wanfang Database（万方数据库）** | | |
| **Number** | **Search strategy** | **Searching results** |
| #1 | 题名或关键词:((“肿瘤” or “癌症” or “癌” or “恶性肿瘤” or “瘤”) and （“养育忧虑” or “养育” or “抚养” or “忧虑” or “压力”or “感受” or “体验”） and（“质性研究” or“质性访谈” or“定性研究” or“质性” or“定性” or“现象学” or“扎根理论” or“描述性分析” ） ) | 875 |
| **10. China Science and Technology Journal Database（维普数据库）** | | |
| **Number** | **Search strategy** | **Searching results** |
| #1 | M=（肿瘤 OR 癌症 OR 癌 OR 恶性肿瘤 OR 瘤） AND M=（养育忧虑 OR 养育 OR 抚养 OR 忧虑 OR 压力 OR 感受 OR 体验） AND M=（质性研究 OR 质性访谈 OR 质性研究 OR 质性 OR 定性 OR 现象学 OR 扎根理论 OR 描述性分析） | 469 |
